# Psychological Medicine

**Published:** 1844-11

**Authors:** 


*Psychological Medicine.*—Prof. Otto has lately written on the "Action of different Medicines on the Mental Faculties." He says that ammonia, opium, musk, castor, wine, ether, and the preparations of gold, enliven the imaginative powers, and render the mind more fertile and creative, while the empyreumatic oils, iodine, arsenic, belladonna, and conium, are apt to induce a tendency to melancholy, and to diminish the energy of the intellectual powers.—*Ibid.*

